# Impact of Demographics on Early-Stage vs. Stage IV Diagnosis in Amelanotic Melanoma: An Analysis of the National Cancer Database

**DOI:** 10.7759/cureus.69398

**Published:** 2024-09-14

**Authors:** Mohammed Al Kurnas, Madison Webster, Xinxin Wu, Peter T Silberstein

**Affiliations:** 1 Medicine, Creighton University School of Medicine, Omaha, USA; 2 Oncology, Creighton University School of Medicine, Omaha, USA

**Keywords:** cutaneous malignant melanoma, metastatic skin cancer, late diagnosis, early identification and diagnosis, health disparity, demographic factors, ­skin cancer, amelanotic melanoma

## Abstract

Introduction

Amelanotic melanoma (AM) is a rare form of melanoma that lacks pigment. Although curable when diagnosed early, it is often missed or mistaken for other benign conditions. There has not been a study investigating the impact of demographic features on the diagnosis of stage 0-I (early-stage) versus stage IV AM.

Objective

This study addresses a gap in knowledge regarding demographic factors that influence the odds of early-stage vs. stage IV diagnosis of AM.

Methods

This study identified 684 patients from the National Cancer Database who were diagnosed with early-stage AM or stage IV AM from 2004 to 2020 and compared them based on age, sex, race, insurance, income, education, insurance status, rurality, facility type, geographic region, and Charleson-Deyo score. Socioeconomic and demographic features of patients with early-stage and stage IV were compared using the chi-squared test, the independent t-test, and multivariate logistic regression. Statistical significance was set at α = 0.05.

Results

Most cases analyzed were White (98.5%), male (57.7%), and lived in a metropolitan setting (86.7%). Males made up most of the early-stage and stage IV groups (55.0% vs. 45% and 66.5% vs. 33.5%, respectively, p < 0.05). Younger age is associated with decreased odds of stage IV disease (OR = 0.973, 95% CI = 0.952-0.993, p < 0.05). In addition, the female sex is associated with decreased odds of stage IV disease (OR = 0.584, 95% CI = 0.381-0.897, p < 0.05).

Conclusions

Age and sex are two variables that influence the odds of stage IV diagnosis in patients with AM, which is strongly associated with worse survival outcomes.

## Introduction

Amelanotic melanoma (AM) is a rare cutaneous subtype of melanoma characterized by the absence of pigment [1,2]. It comprises roughly 2-8% of all melanoma cases [3]. AM may lack the asymmetry and peripheral pigmentation changes characteristically seen in other melanoma subtypes, presenting a clinical challenge [4]. AM also lacks clear diagnostic criteria and can also mimic other skin diseases. Thus, it is routinely diagnosed after a complete workup, including histopathology and immunohistochemistry. Due to delayed diagnosis, AM often progresses into an invasive disease [5]. Prompt diagnosis is essential to prevent unnecessary treatments and avoid misdiagnosis.

Compared to melanotic melanoma (MM), the survival rates for AM are typically lower due to diagnostic difficulty and late stage at diagnosis. According to a previous National Cancer Database (NCDB) study, the five-year and 10-year survival rates for patients with AM are approximately 58.8% and 45.1%, respectively [6]. In MM, the five-year and 10-year survival rates are higher at 75.4% and 62.4%, respectively [6]. Tumor characteristics can serve as prognostic factors for AM, including higher stage diagnosis, increased patient age, tumor location in sun-exposed areas, and increased Breslow thickness [7-10].

Our preliminary studies have supported a significant difference in overall survival between stage I (mean = 118.7 months) and stage IV AM (mean = 42.4 months, p < 0.001) [11]. With long-term survival and effective treatment relying on early diagnosis, it is necessary to understand the socioeconomic factors associated with stage IV diagnosis in AM to assist in the development of methods for early detection and to improve long-term survival. While there have been studies that analyzed the effect of socioeconomic disparities on survival in AM, there are a limited number of studies that have analyzed the demographic and socioeconomic profiles of affected patients and their effect on delayed diagnosis [6-10]. The goal of this study is to identify any significant socioeconomic and demographic factors that have an association with delayed diagnosis.

This article was previously presented as a meeting abstract at the 2023 Association of VA Hematology/Oncology (AVAHO) annual meeting.

## Materials and methods

This retrospective cohort study analyzed patients diagnosed with histologically confirmed AM between 2004 and 2020 sourced from the NCDB with ICD-O-3 code 8730. The NCDB, jointly sponsored by the American College of Surgeons and the American Cancer Society, compiles information from over 1,500 Commission on Cancer-accredited facilities and covers more than 70% of new cancer diagnoses in the United States and Puerto Rico. De-identified patient data was granted through the Participant User Data Files program.

NCDB analytical staging was used to determine patients diagnosed with early-stage (0-I) versus stage IV AM. This stage group uses the pathologic stage group and the clinical stage group when the former is unavailable. Exclusion criteria included missing data, and data with less than 10 cells were suppressed per Cancer Quality Improvement Program guidelines.

Demographic factors analyzed include age, sex, race, insurance, income, education level, population density, facility type, geographic region, great circle distance, and Charlson-Deyo comorbidity score. Cases with missing data were excluded for a final cohort of 684 patients. Race was categorized into two groups due to limited data: White and Other. The “Other” category included subgroups such as African-American, American Indian, Aleutian or Eskimo, Chinese, Japanese, Filipino, Hawaiian, Korean, Vietnamese, Kampuchean, Asian Indian or Pakistani NOS, Asian Indian, Micronesian NOS, Other Asian, Asian NOS, Oriental NOS, and Pacific Islander NOS. Insurance status was determined by the primary payor at diagnosis and categorized into two groups: Private versus Other. “Other” included no insurance, Medicare, Medicaid, and other government insurance. The income quartile was determined by median household income determined by the 2016-2020 census in the patient’s zip code of residence at the time of diagnosis. Education was measured into quartiles by the percentage of residents in the patient’s zip code who did not have a high school degree based on the 2016-2020 census. Population density encompassed metropolitan, urban, and rural designations by using the typology published by the USDA Economic Research Service. Treatment facilities were categorized into academic centers versus non-academic centers. Regions of treatment were categorized into Northeast, South, Midwest, and West based on divisions set forth by the U.S. Bureau. The great circle distance was measured as the distance in miles between the patient’s residence and the treatment hospital. Comorbidities were assessed using the Charlson-Deyo score, and patients were divided into groups with scores of 0, 1, and ≥2.

To assess the differences in the stages of disease (early stage vs. IV) at the time of diagnosis, we employed a chi-square test of independence or independent samples t-tests to examine relationships between categorical variables or continuous variables, respectively. Specifically, the chi-square test was used to determine if there was a significant association between the stage of disease and various categorical predictors.

A binary logistics regression model was employed to identify independent prognostic factors leading to a later stage of diagnosis. The variables included in the multivariable Cox model were age, sex, race, insurance, income, education, insurance status, rurality, facility type, geographic region, and Charleson-Deyo score.

All statistics were conducted using IBM SPSS Statistics for Windows, Version 29.0.2.0 (Released 2023; IBM Corp., Armonk, NY, USA). p < 0.05 indicated statistical significance. The study received an exemption from the Creighton University Institutional Review Board (IRB) under submission number 2004375-01, as the NCDB uses de-identified data.

## Results

Of the 684 cases identified, 520 (76%) cases were either stage 0 or stage I. The average age of an individual diagnosed with stage IV AM was younger than one diagnosed with early-stage disease (63.7 years (SD ± 13.9) versus 66.1 years (±13.9)), but the difference was not statistically significant (p = 0.052) on independent t-testing. Males predominated in both early and stage IV groups. The stage IV group had a slightly higher male predominance than the early-stage group (66.5% vs. 55%). Those with stage IV diagnosis were less likely to have private insurance (42.1%) and more likely to have non-private insurance (57.9%) compared to those with an early-stage diagnosis (p = 0.002). Those with an income in the highest quartile were more likely to be diagnosed with AM compared to those who had lower incomes, but a bigger proportion of those in the highest income quartile were diagnosed with early-stage disease compared to stage IV disease. Similarly, those in the highest education quartile had a larger representation in the early-stage group (37%) compared to those with lower education levels (p = 0.005). However, those in the stage IV group varied more in education levels. Both groups were more likely to be diagnosed at a non-academic center (p = 0.02). In addition, both early and stage IV diagnoses occurred more commonly in the South (p = 0.002). The independent t-test results are listed in Table 1.

**Table 1:** Independent t-test results for early-stage vs. stage IV AM AM, amelanotic melanoma

Variable	Overall	Stage 0-I	Stage IV	p-Value
	Mean (±SD)	Mean (±SD)	Mean (±SD)	
Age (years)	65.6 (±13.9)	66.1 (±13.9)	63.7 (±13.9)	0.052
Great circle distance (miles)	26.9 (±103.8)	29.2 (±118.2)	19.7 (±19.7)	0.333

The distance traveled from residence to the treatment site, the patient’s race, population density, and comorbidity index did not differ significantly between those diagnosed with early or stage IV AM. It is important to note that the vast majority of patients were White, resulting in insufficient representation of other racial groups in statistical analysis. The demographic profiles of patients diagnosed with early-stage and stage IV AM are listed in Table 2.

**Table 2:** Frequencies and chi-square comparison of patients with early-stage and stage IV AM at diagnosis ** Cells with fewer than 10 entries, and those that could reveal such cells, are suppressed. AM, amelanotic melanoma

Variable	Overall (% n)	Stage 0-I (%)	Stage IV (%)	p-Value
Sample size (n)	684	520 (76)	164 (24)	
Sex				0.01
Male	395 (57.7)	286 (55)	109 (66.5)	
Female	289 (42.3)	234 (45)	55 (33.5)	
Race				0.444
White	678 (98.5)	**	**	
Other	10	**	**	
Insurance				
Private	303 (44.3)	234 (45.0)	69 (42.1)	
Other	381 (55.7)	286 (55.0)	95 (57.9)	
Zip code-level median household income (2020 US dollars)				0.002
Less than $46,277	45 (7.4)	26 (5.6)	19 (12.8)	
$46,277-$57,856	97 (15.9)	65 (14.1)	32 (21.6)	
$57,857-$74,062	148 (24.3)	115 (25)	33 (22.3)	
$74,063 or more	318 (52.3)	254 (55.2)	64 (43.2)	
Zip code-level education (2016-2020, % no high school diploma)				0.005
15.3% or more	62 (10.2)	42 (9.1)	20 (13.4)	
9.1-15.2%	143 (23.5)	97 (23.1)	46 (30.9)	
5.0-9.0%	199 (32.7)	151 (32.8)	48 (32.2)	
Less than 5.0%	205 (33.7)	170 (37.0)	35 (23.5)	
Population density				0.937
Metropolitan	593 (86.7)	451 (86.7)	142 (86.6)	
Urban	72 (10.5)	**	**	
Rural	19 (2.8)	**	**	
Facility type				0.02
Academic	270 (39.5)	218 (41.9)	52 (31.7)	
Non-academic	414 (60.5)	302 (58.1)	112 (68.3)	
Geographic location				0.002
Northeast	145 (22.3)	123 (24.7)	22 (14.3)	
South	220 (33.8)	151 (30.4)	69 (44.8)	
Midwest	109 (16.7)	81 (16.3)	28 (18.2)	
West	177 (27.2)	142 (28.6)	35 (22.7)	
Charlson-Deyo score				0.095
0	559 (81.7)	434 (83.5)	125 (76.2)	
1	89 (13.0)	60 (11.5)	29 (17.7)	
≥2	36 (5.2)	26 (5.0)	10 (6.1)	

On multivariate analysis, only age and sex were found to significantly impact the odds of stage IV diagnosis. Younger patients had a slightly lower chance of being diagnosed with stage IV disease (OR = 0.973), and females were also less likely to be diagnosed with stage IV AM (0.584). No other variables included in the analysis demonstrated a statistically significant effect. When controlling for confounding variables and possible interaction effects, age became significant in multivariate analysis, although it was initially not significant on independent t-testing. The multivariate analysis results can be found in Table 3.

**Table 3:** Multivariate analysis of demographic and socioeconomic factors for stage IV diagnosis * Reference: The reference variable is the variable against which all variables are compared to calculate odds. The odds of the event (stage IV) occurring for other variables are calculated relative to the reference variable.

Variable	OR	95% CI: lower bound	95% CI: lower bound	p-Value
Age at diagnosis	0.973	0.952	0.993	0.01
Great circle distance (miles)	0.999	0.994	1.003	0.536
Sex				
Male	*Reference	-	-	-
Female	0.584	0.381	0.897	0.014
Race				
White	*Reference	-	-	-
Other	0.383	0.04	3.671	0.406
Facility type				
Academic center	*Reference	-	-	-
Non-academic center	1.197	0.761	1.881	0.437
Geographic location				
Northeast	*Reference	-	-	-
South	1.718	0.93	3.172	0.084
Midwest	1.576	0.777	3.195	0.208
West	0.965	0.502	1.851	0.913
Insurance				
Private	*Reference	-	-	-
Other	1.285	0.764	2.159	0.345
Median household income quartiles 2016-2020				
Less than $46,277	*Reference	-	-	-
$46,277-$57,856	0.669	0.292	1.533	0.342
$57,857-$74,062	0.437	0.186	1.029	0.058
$74,063 or more	0.474	0.192	1.167	0.104
Percent no high school degree quartiles 2020				
15.3% or more	*Reference	-	-	-
9.1-15.2%	1.23	0.573	2.642	0.595
5.0-9.0%	0.994	0.446	2.215	0.988
Less than 5.0%	0.699	0.289	1.689	0.426
Population density				
Metropolitan	*Reference	-	-	-
Urban	0.481	0.224	1.03	0.06
Rural	0.586	0.145	2.36	0.452
Charlson-Deyo score				
0	*Reference	-	-	-
1	1.632	0.922	2.89	0.093
≥2	1.826	0.767	4.348	0.174

## Discussion

AM is associated with an age of over 65, and this is consistent with the findings of this study, with 66.1 years of age being the average age at diagnosis of AM overall [6,7,9]. This study demonstrated that a younger average age at diagnosis is associated with decreased odds of a stage IV diagnosis of AM. Advanced age was associated with poorer survival in one study, and stage has been shown to be the predominant factor relating to survival in AM [6,7]. With this in mind, our results lend further credence to the importance of early diagnosis. It is important to note that AM is more likely than MM to be misdiagnosed [9]. The rate of misdiagnosis likely influences the increased likelihood of stage IV being diagnosed with increased age. Thus, it is crucial to identify new methods to detect and diagnose AM to decrease the incidence of stage IV diagnosis in patients with advanced age.

Regarding sex, the odds of being diagnosed with stage IV AM increased with male sex. Previous studies were mixed, demonstrating that patients with AM were more often male or female, or that neither sex was associated with an increased likelihood of AM [6-8]. This study is the first to demonstrate that female sex decreases the odds of a stage IV diagnosis. It is theorized that women are more likely to pay more attention to their skin [12]. Women are also more likely to communicate health concerns and have more contact with the healthcare system than men throughout their lives [12]. It is also posited that men have increased chronic sun exposure than women, which may also explain our findings [7]. In addition, females have been found to have double the survival rate as males in MM, with genetic differences being shown to significantly influence survival [13]. The protective effect that the female sex has on stage at diagnosis in AM in this study indicates that this trend in survival may be true for AM as well, considering that stage at diagnosis is the predominant factor determining survival in AM [6,7]. Further studies directly comparing male and female patients could elucidate mechanisms that bestow a protective effect on females to better inform initiatives to decrease the incidence of stage IV disease among men.

The findings of this study can be compared with previous findings by investigator P.S. in an NCDB study on MM. When compared to non-Hispanic white patients, the odds of stage IV MM were increased for all other races [14]. The present study did not find any association between race and the odds of stage IV AM. Most patients with AM in this study and in the literature are White, and race has not been shown to be associated with survival in AM [6,7]. Decreased education is also known to increase the odds of stage IV MM [14]. Patients with MM are more likely to actively self-screen or request a skin examination if they have an understanding of their risk for melanoma [15]. Our study showed no association between stage IV AM and education level. This may indicate that patients are likely unaware of their risk for AM across all education levels and may neglect suspicious lesions that lack pigment, thus leading to a more advanced stage of disease on diagnosis. Increased awareness of AM may lead to an increased likelihood of patients requesting skin examinations for lesions without pigmentation, resulting in an earlier diagnosis of AM.

Timely diagnosis is crucial in achieving better outcomes for patients with AM. Earlier diagnosis leads to improved survival [6,7]. Moreover, there is evidence that AM may be intrinsically more aggressive than MM at baseline. [6,7,9]. This may also contribute to an increased likelihood of stage IV at diagnosis. AM is also more likely to be misdiagnosed clinically and pathologically than MM [9]. Without pigmentation, clinicians more easily misinterpret AM as benign nevi or other skin diagnoses [15]. The findings in this study suggest that greater awareness of amelanotic skin lesions in males can lead to earlier detection, resulting in decreased stage IV diagnosis.

Limitations

This study has limitations, primarily due to its reliance on retrospective data from the NCDB. While the NCDB encompasses about 70% of new cancer diagnoses in the United States, it might not entirely represent the broader population with AM. AM identification is based on ICD or histology codes, so cases that were not biopsied could be missed. Furthermore, data from cancer facilities that are not part of the NCDB registry are excluded. Lastly, logistic regression may only partially account for some potential confounding variables, with some significant findings in chi-square tests not being replicated in the regression analysis.

## Conclusions

This is one of the first studies to compare the demographic features that influence the odds of early-stage AM with stage IV AM. From this NCDB analysis, it can be concluded that female sex and younger age decrease the odds of a stage IV diagnosis. These findings can increase provider awareness when diagnosing lesions suspicious for AM and can provide patients at risk for AM with more information to make informed decisions regarding their health. It may be warranted for clinicians to increase their index of suspicion for AM and to consider more extensive testing when evaluating skin lesions that lack pigment in males. As older males are at an increased risk for stage IV disease, it is important to develop novel strategies for detecting earlier disease to decrease the incidence of stage IV AM in this population and thereby minimize the disparity in outcomes between males and females.
